# hvEEGNet: a novel deep learning model for high-fidelity EEG reconstruction

**DOI:** 10.3389/fninf.2024.1459970

**Published:** 2024-12-20

**Authors:** Giulia Cisotto, Alberto Zancanaro, Italo F. Zoppis, Sara L. Manzoni

**Affiliations:** ^1^Department of Informatics, Systems and Communication, University of Milano-Bicocca, Milan, Italy; ^2^Department of Information Engineering, University of Padova, Padova, Italy

**Keywords:** EEG, VAE, variational autoencoder, latent representation, motor imagery

## Abstract

**Introduction:**

Modeling multi-channel electroencephalographic (EEG) time-series is a challenging tasks, even for the most recent deep learning approaches. Particularly, in this work, we targeted our efforts to the high-fidelity reconstruction of this type of data, as this is of key relevance for several applications such as classification, anomaly detection, automatic labeling, and brain-computer interfaces.

**Methods:**

We analyzed the most recent works finding that high-fidelity reconstruction is seriously challenged by the complex dynamics of the EEG signals and the large inter-subject variability. So far, previous works provided good results in either high-fidelity reconstruction of single-channel signals, or poor-quality reconstruction of multi-channel datasets. Therefore, in this paper, we present a novel deep learning model, called hvEEGNet, designed as a hierarchical variational autoencoder and trained with a new loss function. We tested it on the benchmark Dataset 2a (including 22-channel EEG data from 9 subjects).

**Results:**

We show that it is able to reconstruct all EEG channels with high-fidelity, fastly (in a few tens of epochs), and with high consistency across different subjects. We also investigated the relationship between reconstruction fidelity and the training duration and, using hvEEGNet as an anomaly detector, we spotted some data in the benchmark dataset that are corrupted and never highlighted before.

**Discussion:**

Thus, hvEEGNet could be very useful in several applications where automatic labeling of large EEG dataset is needed and time-consuming. At the same time, this work opens new fundamental research questions about (1) the effectiveness of deep learning models training (for EEG data) and (2) the need for a systematic characterization of the input EEG data to ensure robust modeling.

## 1 Introduction

High-fidelity reconstruction of electroencephalography (EEG) data is of key relevance to many deep learning (DL) tasks for EEG analysis, such as anomaly detection, automatic labeling, classification, and brain-computer interface control (Kodama et al., 2023; Beraldo et al., 2022). The most significant open challenges for the DL models are the complex dynamics of the EEG signals and the large inter-subject variability. So far, these two aspects have prevented DL to offer a gold-standard for high-accurate reconstruction of multi-channel EEG data (Lotte et al., 2018). A critical issue for DL methods is the dependency on the training set, as Gyori et al. (2022) recently pointed out in the domain of magnetic resonance imaging data: a training dataset of poor quality, as well as a training set distributed in a non-representative way might induce biases in the trained model and, consequently, lead to poor results in the task the model is expected to perform e.g., classification or anomaly detection. At the same time, in the neuroscience domain, it is very common to meet this situation (Pion-Tonachini et al., 2019), as the complex dynamics of any biological signal and the large inter-individual variability make it difficult to obtain a clean, large, and representative training set.

Nonetheless, in this paper, we present a novel deep learning model, called *hvEEGNet*, designed as a hierarchical variational autoencoder (VAE), where the encoder and the decoder modules have been inspired by the popular EEGNet architecture (Lawhern et al., 2016). The proposed model was trained by using a novel loss function based on dynamic time warping (DTW), never used before for EEG data, but very well-suited for time-series (Bankó and Abonyi, 2012). We show that the combination of this specific architecture and the training strategy brings to high-fidelity reconstruction of multi-channel EEG signals. Interestingly, this is also consistent across different subjects. To test our model, we use a benchmark dataset called *dataset 2a* to have a fair comparison with previous works. Then, we deepen the investigation of the model's training over this particular dataset, and we empirically observe that there is a relationship between the training time (i.e., the number of epochs needed to train the model for each subject) and the particular distribution of the EEG values in each subject. We show that 80 epochs are enough to obtain almost perfect reconstruction for all subjects in the dataset, but for some of them 20 epochs are sufficient. Thus, with this work, we shed light on the importance of conducting a systematic analysis of the input EEG data and its variability across different subjects to ensure proper DL models training, a step never reported by previous works (as far as the authors know).

In this paper, we provide the following novel contributions:

1. we show that the choice of DTW as the loss function, in place of the more standard mean square error (MSE), has a key role in the accurate EEG reconstruction;
2. a simple VAE model cannot properly recover multi-channel EEG data, while a hierarchical architecture (i.e., *hvEEGNet*) provides high-fidelity reconstruction;
3. *hvEEGNet* is trained very fastly, in a few tens of epochs, despite the small size of the training dataset;
4. finally, using the trained model (in a within-subject modality), we discovered some significant instrumental anomalies in the benchmark dataset (never pointed out before).

## 2 State of the art

Reconstructing a multi-channel EEG dataset with high-fidelity is a challenging task, given the typical very low signal-to-noise ratio characterizing EEG signals (Cisotto, 2021), the fast dynamics of each signal and its relationship with signals acquired from different locations of the scalp, and the large inter-subject variability (both between healthy and patients, but also across different individuals sharing the same condition). So far, even with the development of DL techniques, there is no gold-standard model available to obtain a general-purpose high-fidelity EEG reconstruction. From the most recent literature, two main trends can be highlighted: on one side, some recent works proposed DL models to reconstruct *multi-channel* EEG, but they can only achieve poor reconstruction quality. For example, Bethge et al. (2022) proposed EEG2VEC, i.e., a VAE architecture to encode emotions-related EEG signals in the VAE latent space. The authors succeeded in reconstructing the low-frequency components of the original EEG signals but the higher frequency ones were not properly recovered. Moreover, the output signals appeared to be largely attenuated (amplitudes often halved w.r.t. the original ones). According to the authors' explanation, this was due to the particular design of the decoder which might have introduced aliasing and artifacts. In one of the our previous work (Zancanaro et al., 2023a), we also found similar results. There, we proposed *vEEGNet-ver1*, a new VAE architecture designed to extract latent representations of multi-channel EEG both to classify EEG signals [via feed-forward neural network (FFNN)] and to reconstruct them. We achieved state-of-the-art classification performance, but only poor reconstruction: specifically, we were able to retrieve only a slow frequency component [related to the initiation of the movement (Bressan et al., 2021; Ofner et al., 2019)] but we failed to recover higher frequencies information. On the other side, other works (Al-Marridi et al., 2018; Dasan and Gnanaraj, 2022; Khan et al., 2023; Liu et al., 2020) were able to reconstruct EEG signals with high accuracy but from a *single-channel* acquisition setup. They mostly dealt with single-channel reconstruction with the perspective of compression in wireless portable devices. In Al-Marridi et al. (2018), the authors implemented a convolutional autoencoder to compress EEG signals, showing good abilities to reconstruct single channel EEG signals with a relatively high compression ratio (up to 98% with distortion of 1.33%). Dasan and Gnanaraj (2022) proposed a multi-branch denoising autoencoder to compress EEG signals coming from one only sensor, together with the Electrocardiogram (ECG) and electromyographic (EMG) signals coming from other two sensors with the purpose to ensure continual learning (continuous fine-tuning) and real-time health monitoring. Each signal modality (EEG, Electrocardiogram (ECG), electromyographic (EMG)) was independently pre-processed, and then the autoencoder provided a multi-modal latent representation. The results showed a good trade-off between compression ratio and reconstruction quality over three public datasets. This work represents an interesting approach, but it assumes to have complementary information about the subjects (their muscular and heart activity). Also, this approach can be adopted in those applications where movement is involved, but it might be more difficult to apply with pure cognitive tasks (e.g., imagination of the movement or sleeping). Lastly, it might also be expected that the use of portable devices bring lower EEG signal quality (typically capturing lower frequencies), thus making the final recovery easier. Finally, Khan et al. (2023) used a shallow autoencoder with a low dimensional latent space (8–64) to classify single-channel EEG signals of a public dataset into epileptic vs. healthy classes, achieving 97% accuracy, sensitivity and specificity over 96%. They also achieved very good reconstruction, as shown in two representative signals. Unfortunately, the authors did not report the power spectrum of the original EEG signals, thus making it difficult to fully ensure a reproducibility of these good performance on other, more complex (i.e., with larger bandwidth), EEG data. In Liu et al. (2020), an autoencoder was trained within a larger deep learning architecture with the goal of compressing and reconstructing the EEG data. Particularly, its input is given by the output of a convolutional neural networks (CNN), used as feature extractor. The CNN and the autoencoder were trained together. Then, the optimized features were fed to a FFNN that operated a classification (with separate training). They tested their model on the very popular DEAP (Koelstra et al., 2012) and SEED (Zheng and Lu, 2015) public emotions-related EEG datasets, reaching satisfactory results (accuracy around 90% in both arousal and valence categories for DEAP, and over 96% for the three classes of SEED). However, the authors did not provide results about the quality of the reconstruction of the autoencoder. It can be said that most literature is focused on using DL techniques to solve application-tailored classification problems. However, a systematic investigation to extract more general conclusions on the reconstruction effectiveness of those models is still lacking.

Similar considerations hold true about the application of DL for the detection of anomalies in EEG, a natural related application of high-fidelity reconstruction. The vast majority of the studies at the state-of-the-art have targeted the automatic identification of pathological events (e.g., seizures) w.r.t. certified, artifact-free, and healthy EEG signals. In this task, autoencoders have been largely and successfully employed, as they offer the inherent possibility to be used as anomaly detectors (Pang et al., 2021), provided that they can learn from *good quality* training data. As an example, Emami et al. (2019) showed that a model based on an autoencoder and a threshold labeling system can detect epileptic seizures with 100% accuracy in about 92% cases (22 subjects over 24 of a private dataset). Ortiz et al. (2020) reached similar outstanding results (accuracy of 96%, sensitivity of 86%, specificity of 100%, area under the curve (AUC) of 92%) using a cascaded system with an autoencoder trained to reconstruct the time-series of some handcrafted features to enhance the spatial differences in the brain activity of patients suffering from dyslexia and healthy controls [a support vector machine (SVM) later classifies the two classes].

Nevertheless, there is no well-established definition of *normality* for EEG signals, as much as it is fairly difficult to have a certified, large, and artifact-free dataset (even in the case of healthy subjects) (Gabardi et al., 2023). Therefore, it would be more realistic to train an autoencoder model on a mixture of clean and noisy data, in line with some other literature (not necessarily addressing biological data). For instance, in Zhou and Paffenroth (2017), the authors proposed a *robust autoencoder*, i.e., a combination of a robust PCA (RPCA) and an autoencoder, where the autoencoder was used for data projection in the (reduced) principal components space (in place of the usual linear projection). Unfortunately, there is a limited literature on this kind of autoencoders, as confirmed by a recent survey (Al-amri et al., 2021). In Xing et al. (2020), the authors proposed a combination of an evolving spiking neural network and a Boltzmann machine to identify anomalies in a multimedia data stream. The proposed training algorithm was able to localize and ignore any random noise that could corrupt the training data. In Dong and Japkowicz (2018), a model composed by an ensemble of autoencoders was employed to identify anomalies in data streams. The authors claimed that the training algorithm made the presence of noisy samples in the training data not statistically significant, thus ensuring model's robustness to noise. In Qiu et al. (2019), an architecture made by the sequence of a CNN, an long-short term memory (LSTM), a FFNN, and a softmax layer was proposed to identify anomalies. Interestingly, a VAE was preliminarily used to over-sample the dataset, before training the classifier (i.e., the FFNN with the softmax layer). The model was tested on the AIOps-KPI public dataset (Li et al., 2022), achieving an accuracy of 77% (KP1), 75% (KP2), 83% (KP3), and 75% (KP4). Nevertheless, to the best of our knowledge, this kind of approaches has never been applied to EEG data, yet.

Thus, some fundamental open challenges emerge from the state of the art (SOTA) review to be solved, including (1) the high reconstruction errors or the generation of traces that are not faithful to the original signals, (2) the lack of focus on the reconstruction task (in favor of classification or anomaly detection) even if the architecture has this possibility, and (3) no rigorous investigation on the impact of the input quality and distribution on the training of DL models. Thus, in this paper we shed light on these important challenges in relation to high-fidelity reconstruction of multi-channel EEG data.

## 3 Materials and methods

In this section, we present the basic modules as well as the overall architecture of our proposed model, *hvEEGNet*. Also, to support our design choices, we introduce another model, namely *vEEGNet-ver3*, which shares the same new loss function with hvEEGNet, but a different (simpler, i.e., not hierarchical) architecture. Furthermore, we describe the metrics and the methodologies we employed to evaluate our models.

### 3.1 Variational autoencoder

The common overall architecture of our both models is the VAE.

Unlike traditional autoencoders, i.e., producing a deterministic encoding for each input, VAE is able to learn a probabilistic mapping between the input data and a latent space, which is additionally learned as a structured latent representation (Kingma and Welling, 2013, 2019). Given the observed data **x** and assuming **z** to be the latent variables, with a proper training, a VAE learns the variational distribution *q*_ϕ_(**z**|**x**) as well as the generative distribution *p*_θ_(**x**|**z**), using a pair of (deep) neural networks (acting as the encoder and the decoder), parameterized by ***ϕ*** and ***θ***, respectively (Blei et al., 2017). The training loss function, denoted as L_*VAE*_, accounts for the sum of two different contributions: the Kullback-Leibler divergence between the variational distribution *q*_ϕ_(**z**|**x**) and the posterior distribution *p*_θ_(**x**|**z**), denoted as L_KL_, and the reconstruction error, denoted as L_*R*_, which forces the decoded samples to approximate the initial inputs. Thus, the loss function adopted for the VAE is


(1) $$\begin{array}{l} L_{VAE}=L_{KL}+L_{R}=-KL\left[q_{\phi }\left(\text{z}|\text{x}\right)||p\left(\text{z}\right)\right]+{𝔼}_{q}\left(\log p_{\theta }\left(\text{x}|\text{z}\right)\right) \end{array}$$

Assuming normal distribution as a prior for the sample distribution in the latent space, it is possible to rewrite Equation 1 as follows


(2) $$\begin{array}{l} L_{VAE}=-\frac{1}{2}\overset{d}{\underset{i=1}{\sum }}\left(\sigma_{i}^{2}+\mu_{i}^{2}-1-log\left(\sigma_{i}^{2}\right)\right)+E_{q}\left(\log p_{\theta }\left(\text{x}|\text{z}\right)\right) \end{array}$$

where μ_*i*_ and $\sigma_{i}^{2}$ are the predicted mean and variance values of the corresponding *i*-th latent component of **z**.

In this work, we adopted this basic architecture to propose vEEGNet *version 3* (vEEGNet-ver3). The details characterizing this specific model are reported in Section 3.3.

### 3.2 Hierarchical VAE

A hierarchical VAE (Vahdat and Kautz, 2021) is the evolution of a standard VAE enriched by a hierarchical latent space, i.e., multiple layers implementing a latent space each. In fact, standard VAEs suffer from the lack of accuracy in details reconstruction, given by the trade-off between the reconstruction loss and the Kullback-Leibler divergence contributions, thus generating the tendency to generate slightly approximated data (e.g., blurred images), only. Hierarchical VAEs attempt to solve this problem by using multiple latent spaces, where each of them is trained to encode different levels of detail in the input data. Assuming a model with *L* latent spaces, its loss function can be written as


(3) $$\begin{array}{l} L_{HVAE}=L_{KL}+L_{R} \end{array}$$

where


(4) $$\begin{array}{l} L_{KL}=-KL\left[q_{\phi }\left({\text{z}}_{1}|\text{x}\right)||p\left({\text{z}}_{1}\right)\right]-\overset{L}{\underset{l=2}{\sum }}KL\left[q_{\phi }\left({\text{z}}_{\text{l}}|\text{x},{\text{z}}_{<l}\right)||p\left({\text{z}}_{\text{l}}|{\text{z}}_{<l}\right)\right], \end{array}$$

with $q_{\phi }\left({\text{z}}_{\text{l}}|,{\text{z}}_{<l}\right)=\prod_{i=1}^{l-1}q_{\phi }\left({\text{z}}_{\text{l}}|\text{x},{\text{z}}_{\text{<i}}\right)$ as the approximate posterior up to the (*l*−1) level and the conditional in each prior *p*(*z*_*l*_|*z*_<*l*_) and approximate posterior *q*_ϕ_(**z**_**l**_|**x**, **z**_** < i**_) is represented as a factorial normal distribution. The notation is taken from the original paper (Vahdat and Kautz, 2021). Particularly, the symbol **z**_<*i*_ means that the random variable is conditioned by the output of all latent spaces from 1 to *i*.

In this work, we adopted this basic architecture to propose *hierarchical* vEEGNet (hvEEGNet). The details characterizing this specific model are reported in Section 3.5.

### 3.3 vEEGNet-ver3

**Figure 1:**
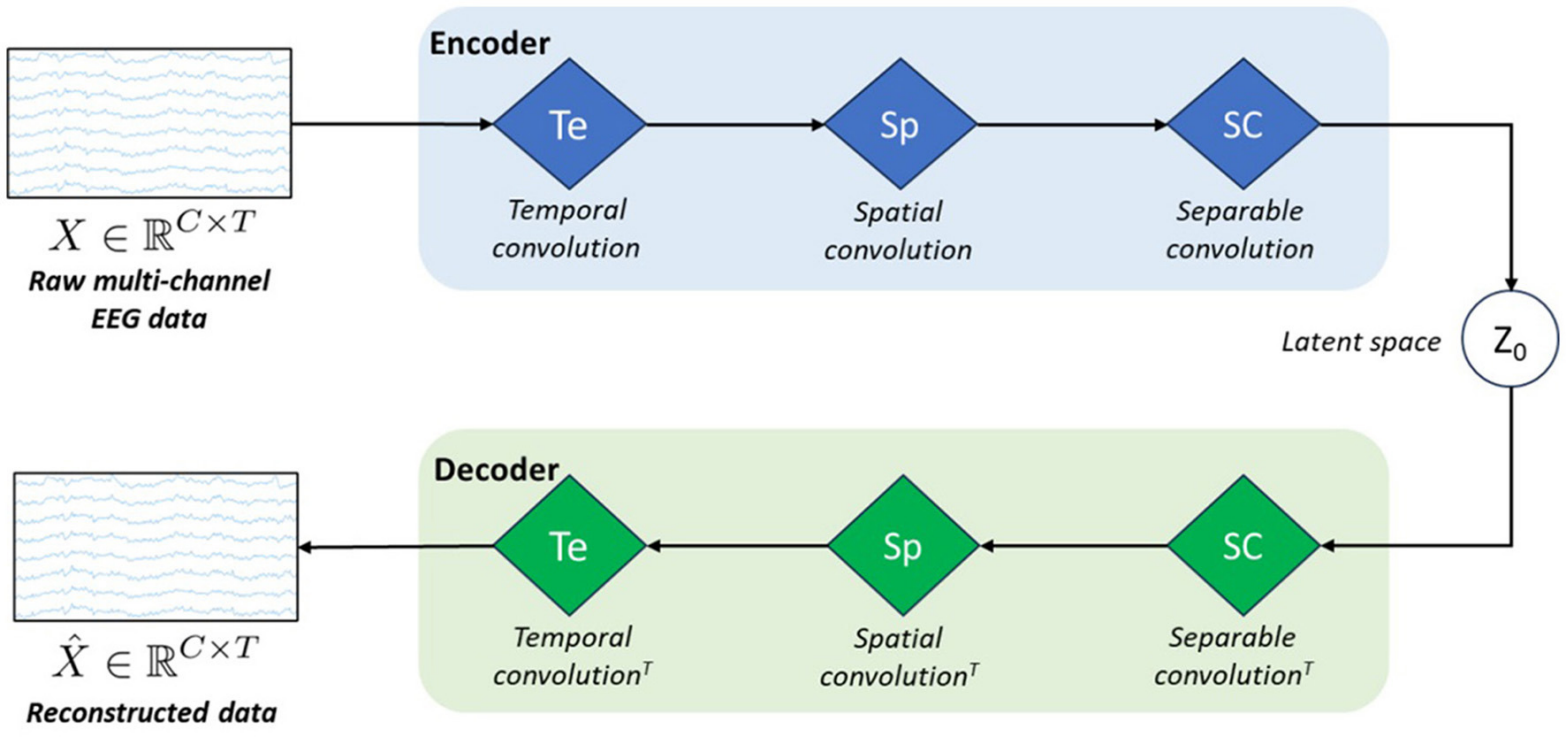
Schematic architecture of our model called *vEEGNet-ver3*. The encoder block is formed by three blue diamonds representing three different processing layers: i.e., Te stands for *temporal convolution*, Sp stands for *spatial convolution*, and SC stands for *separable convolution*. The decoder block includes three green diamonds representing the same operations, in the reverse order and using the transpose convolution (^*T*^). *z*_0_ represents the latent space.

Figure 1 represents the schematic architecture of this simple VAE model. As any conventional VAE, it consists of an encoder, a latent space, and a decoder.

However, inspired by the work of Lawhern et al. (2016), we designed the encoder as the popular EEGNet architecture, i.e., with the three processing blocks: in the first block, a horizontal convolution (that imitates the conventional temporal filtering) is followed by a batch normalization. In the second block, a vertical convolution, acting as a spatial filter, is applied. This operation is then followed by an activation and an average pooling step. The third, and last, block performs a separable convolution with a horizontal kernel, followed by an activation and an average pooling step. We always used, as in Lawhern et al. (2016), the exponential linear unit (ELU) activation function. At the output of the third block, the obtained *D*×*C*×*T* tensor is further transformed by means of a sampling layer which applies a convolution with a 1 × 1 kernel, thus doubling its depth size, resulting in a 2*D*×*C*×*T* tensor. Finally, the latter is projected onto the latent space (i.e., of dimension *N* = *D*·*C*·*T*). In line with other previous works (Zancanaro et al., 2023a; Kingma and Welling, 2013), the first *N* elements of the depth map were intended as the marginal means (***μ***) and the second *N* elements as the marginal log-variances (***σ***) of the Gaussian distribution represented in the latent space. Then, to reconstruct the EEG data, the latent space **z**_**0**_ is sampled using the reparameterization trick, as follows:


$${\text{z}}_{0}=\mu +\sigma \cdot N\left(0,1\right),$$

where N(**0**, **1**) is standard multivariate Gaussian noise (with dimension *N* = *D*·*C*·*T*).

Finally, in the projection onto the latent space, we apply a 1 × 1 convolution to the output of the encoder, thus obtaining a depth map whose first half is taken as the mean and the second half as the log-variance of the distribution of the latent space.

To note, this architecture is very similar to other previous architectures proposed by the authors in Zancanaro et al. (2023a) and in Zancanaro et al. (2023b). However, it introduces a very significant novelty that leads this new model to perform much better than the older ones. The reconstruction error L_*R*_ of the VAE loss function expressed by Equation 1 was here quantified by the DTW similarity score (Sakoe and Chiba, 1978), i.e., replacing the more standard MSE. DTW leads to a more suitable measure of the similarity between two time-series (Bankó and Abonyi, 2012), thus allowing the model better learn to reconstruct EEG data. In fact, DTW is known to be more robust to non-linear transformations of time-series (Huang and Jansen, 1985), thus capturing the similarity between two time-series even in presence of time shrinkage or dilatation, i.e., warpings. This cannot be achieved by MSE, which is highly sensitive to noise, i.e., the error computed by MSE rapidly increases when small modifications are applied to time-series.

### 3.4 DTW and normalized DTW

In brief, given two time-series *a*(*i*) and *b*(*j*), where *i, j* = 1, 2, ..., *T* (i.e., for simplicity, we consider two series with the same length), DTW is a time-series alignment algorithm that extensively searches for the best match between them, by following a five-step procedure:

1. The cost matrix W is initialized, with each row *i* associated with the corresponding amplitude value of the first time-series *a*(*T*−*i*+1), while each column *j* associated with the corresponding amplitude value of the second time-series *b*(*j*).
2. Starting from position W(0, 0), the value of each matrix element is computed as W(*i, j*) = |*a*(*i*)−*b*(*j*)|+min[W(*i*−1, *j*−1), W(*i, j*−1), W(*i*−1, *j*)], if *i, j*>0, otherwise W(*i, j*) = |*a*(*i*)−*b*(*j*)|.
3. The optimal warping path is identified as the minimum cost path in W, starting from the element W(1, *T*), i.e., the upper right corner, ending to the element W(*T*, 1).
4. the array *d* is formed by taking the values of W included in the optimal warping path. Note that *d* might have a different (i.e., typically longer) length compared to the two original time-series, as a single element of one series could be associated with multiple elements of the other.
5. Finally, the *normalized* DTW score is computed as
6.
$$\begin{array}{l} score=\frac{\sum_{k=1}^{K}d\left(k\right)}{K} \end{array}$$
7. where *K* is the length of the array *d*. To note, normalization was not applied during the models' training (to keep this contribution in the range of the other loss function contributions). Whereas, during the performance evaluation, we used the normalized score. Nevertheless, this difference did not induce criticisms, as all segments share the same length.

### 3.5 hvEEGNet

**Figure 2:**
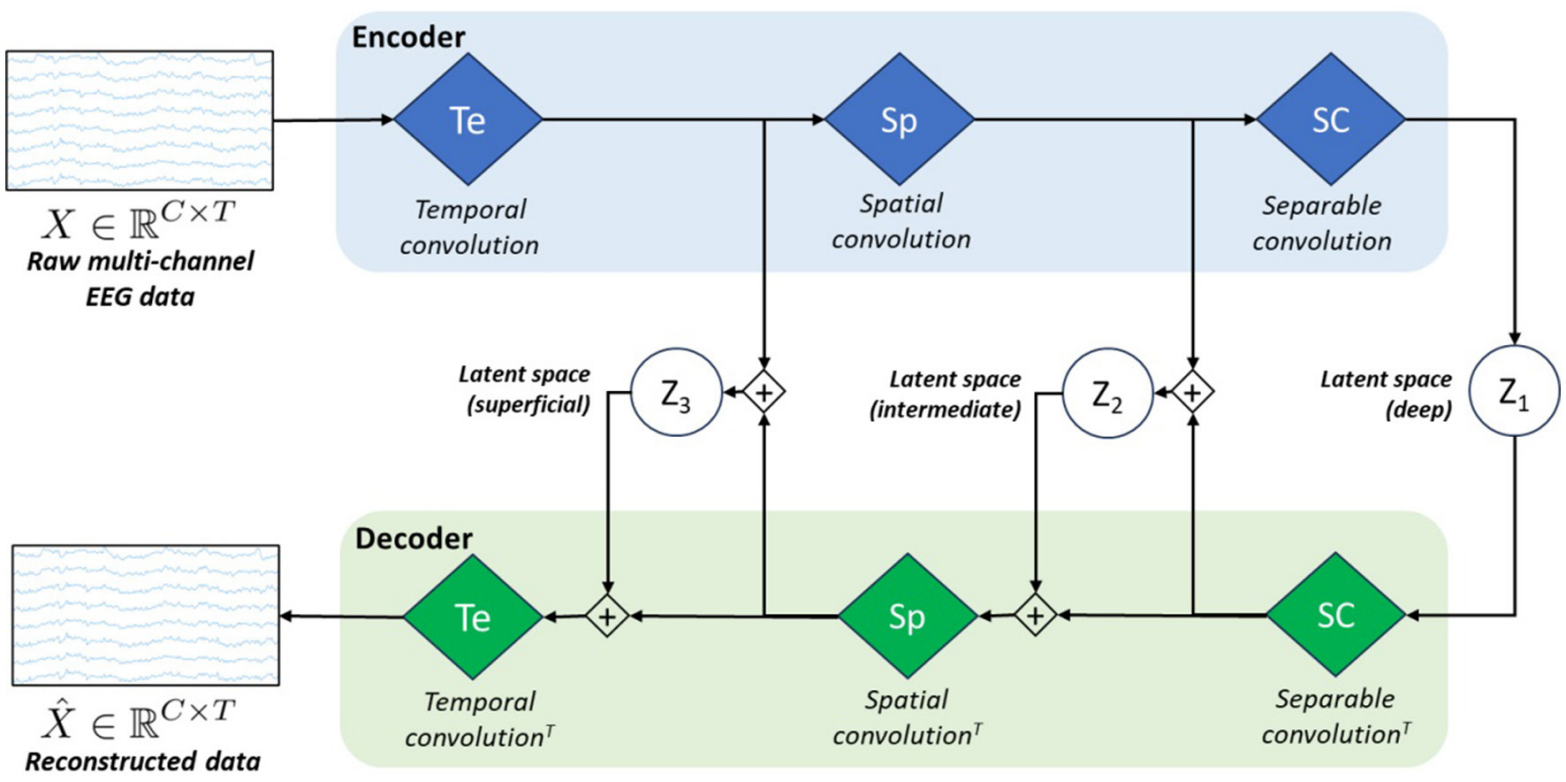
Schematic architecture of our model called *hvEEGNet*. The encoder block is formed by the same three processing layers (i.e., blue diamonds) as in vEEGNet-ver3 (Figure 1). The decoder block includes three green diamonds representing the same operations, in the reverse order and using the transpose convolution (^*T*^). *z*_1_, *z*_2_, and *z*_3_ represent the latent spaces obtained at the three different processing levels.

As we observed sub-optimal reconstruction results with vEEGNet-ver3 and in line with other literature on computer vision (Vahdat and Kautz, 2021), we developed a new architecture, called hvEEGNet, to overcome the issues of vEEGNet-ver3. The most relevant change in hvEEGNet w.r.t. vEEGNet-ver3 is its hierarchical architecture with three different latent spaces, namely *z*_1_, *z*_2_, and *z*_3_, with *z*_1_ being the deepest one. Each of them is located at the output of each main block of the encoder, i.e., after the temporal convolution (Te) block (*z*_3_), after the spatial convolution (Sp) block (*z*_2_), and after the separable convolution (SC) block (*z*_1_). The input to the decoder's Sp block is now given by the linear combination (i.e., the sum) of the SC block's output and the sampled data from *z*_2_. Similarly, the input to the decoder's Te block is obtained by the sum of the Sp block's output and the sampled data from *z*_3_. Incidentally, but significantly, it is worth noting that we kept here using the DTW algorithm to compute the reconstruction loss L_R_ (with reference to Equation 5). The main structure of the model is depicted in Figure 2.

Tables 1 and 2 report the details of both vEEGNet-ver3 and hvEEGNet architectures.

**Table 1:** Parameters' values of the vEEGNet-ver3's and hvEEGNet's encoder, respectively.

		**vEEGNet-ver3**				**hvEEGNet**			
**Parameters name**		**Kernel**	**In. dep**.	**Out. dep**.	**Notes**	**Kernel**	**In. dep**.	**Out. dep**.	**Notes**
First block (temporal filter)	Convolution 2d	(1, 128)	1	8	Depth-wise convolution	(1, 128)	1	8	Depth-wise convolution
	Batch norm 2d	–	–	–	Default parameters	–	–	–	Default parameters
Second block (spatial filter)	Convolution 2d	(1, 22)	8	16	Depth-wise convolution	(1, 22)	8	16	Depth-wise convolution
	Batch Norm 2d	–	–	–	Default parameters	–	–	–	Default parameters
	Activation	- -	–	–	ELU	–	–	–	ELU
	Average pooling	(1, 4)	–	–	–	–	–	–	No pooling used
	Dropout	–	–	–	*p* = 0.5	–	–	–	*p* = 0.5
Third block (separable convolutoin)	Convolution 2d	(1, 32)	16	16	Depth-wise convolution	(1, 32)	16	16	Depth-wise convolution
	Convolution 2d	(1, 1)	16	16	Pointwise convolution	(1, 1)	16	16	Pointwise convolution
	Batch norm 2d				Default parameters				Default parameters
	Activation	–	–	–	ELU	–	–	–	ELU
	Average pooling	(1, 8)	–	–	–	(1, 10)	–	–	–
	Dropout	–	–	–	*p* = 0.5	–	–	–	*p* = 0.5
Sample layer	Convolution 2d	(1,1)	16	32	Pointwise convolution	(1, 1)	16	32	Pointwise convolution

In. dep., input depth; out. dep., output depth.

**Table 2:** Parameters' values of the vEEGNet-ver3's and hvEEGNet's decoder, respectively.

		**vEEGNet-ver3**				**hvEEGNet**			
**Parameters name**		**Kernel**	**In. dep**.	**Out. dep**.	**Notes**	**Kernel**	**In. dep**.	**Out. Dep**.	**Notes**
Third block (separable convolutoin)	Dropout	–	–	–	*p* = 0.5	–	- -	–	*p* = 0.5
	Upsample	(1, 8)	–	–	–	(1, 10)	–	-	–
	Activation	–	–	–	ELU	–	–	–	ELU
	Batch Norm 2d				Default parameters				Default parameters
	Transpose Convolution 2d	(1, 1)	16	16	Pointwise convolution	(1, 1)	16	16	Pointwise convolution
	Transpose Convolution 2d	(1, 32)	16	16	Depth-wise convolution	(1, 32)	16	16	Depth-wise convolution
Second block (spatial filter)	Dropout	–	–	–	*p* = 0.5	–	–	- -	*p* = 0.5
	Upsample	(1, 4)	–	- -	–	–	–	–	No pooling used
	Activation	–	–	–	ELU	–	–	–	ELU
	Batch Norm 2d	–	–	–	Default parameters	–	–	–	Default parameters
	Transpose Convolution 2d	(1, 22)	8	16	Depth-wise convolution	(1, 22)	8	16	Depth-wise convolution
First block (temporal filter)	Batch Norm 2d	–	–	–	Default parameters	–	–	–	Default parameters
	Transpose Convolution 2d	(1, 128)	1	8	Depth-wise convolution	(1, 128)	1	8	Depth-wise convolution

In. dep., input depth; out. dep., output depth.

### 3.6 Outlier identification

As our architectures implement completely self-supervised models, we have the opportunity to use them as anomaly detectors. In line with the vast majority of related work (as introduced in Section 2), in the present study, we define as an *outlier* any sample (i.e., EEG segment) that is very poorly reconstructed. This, in turn, is verified by large values of the DTW similarity score between the reconstructed EEG sample and the original one. To identify such samples, we decided to use the k-nearest neighbors (kNN) algorithm (Cover and Hart, 1967). kNN is an unsupervised machine learning (ML) algorithm that computes the distance between every sample and its *k*-th nearest neighbor (with *k* properly chosen). All samples in the dataset are sorted w.r.t. increasing values of such distance. Those points whose distance (from their *k*-th nearest neighbor) exceeds a user-defined *threshold* are labeled as outliers.

In the second part of this study, we used hvEEGNet model to identify the outliers. Before applying kNN, we performed two pre-processing steps: we computed the DTW similarity score for the EEG segment (i.e., channel- and repetition-wise). To note, by definition (see Equation 5), the score is normalized by the number of time points in the series (even though all time-series have fixed length in this work). For each training run, we built the following matrix **E**:


(5) $$\begin{array}{c} \text{E}\left(t\right)=\left[\begin{array}{cccc} e_{11}\left(t\right) & e_{12}\left(t\right) & \cdots & e_{1C}\left(t\right)\\ e_{21}\left(t\right) & \ddots & \cdots & \vdots \\ \vdots & \vdots & \ddots & \vdots \\ e_{R1}\left(t\right) & \cdots & \cdots & e_{RC}\left(t\right) \end{array}\right] \end{array}$$

with *t* = 1, 2, ..., *T*, given *T* the number of training runs, *r* = 1, 2, ..., *R*, with *R* the number of segments (i.e., task repetitions), and *c* = 1, 2, ..., *C*, with *C* the number of EEG channels. Then, *e*_*rc*_(*t*) represents the normalized DTW value obtained from the reconstruction of the *r*-th segment at the *c*-th channel after training the hvEEGNet model in the *t*-th training run. Finally, the matrices **E**(*t*), with *t* = 1, 2, ..., *T* are averaged to obtain $\bar{\text{E}}$, and then kNN is applied. Also note that kNN took every sample of the dataset as defined by a *C*-dimensional EEG segment (i.e., one row in matrix $\bar{\text{E}}$). This allowed us to identify two types of outliers: (1) repetitions where all (or, the majority of the) channels were affected by some mild to severe problem, or (2) repetitions where only one (or, a few) channel was highly anomalous. Both are very common situations that might occur during neuroscience experiments (Teplan et al., 2002).

### 3.7 Implementation

We employed PyTorch to implement and to design and train our models.

To implement the newly proposed loss function, i.e., including the DTW computation, we exploited the soft-DTW loss function CUDA time-efficient implementation (available at: https://github.com/Maghoumi/pytorch-softdtw-cuda) (Maghoumi, 2020; Maghoumi et al., 2021). In fact, the original DTW algorithm is quite time-consuming and employs a minimum function that is non-differentiable. Then, in Cuturi and Blondel (2017), a modification of the original algorithm was proposed to specifically be used in DL models, i.e., to be differentiable, thus suitable as a loss function. Then, CUDA was employed to make it time-efficient, too. Also, it is worth noting that DTW works with 1D time-series. However, our models aimed to reconstruct multi-channel EEG time-series. Then, during training, we computed the channel-wise DTW similarity score between the original and the reconstructed EEG segment. Then, in the loss function, we added the contribution coming from the sum of all channel-wise DTW scores.

The models were trained using the free cloud service offered by Google Colab, based on Nvidia Tesla T4 GPU. The hyperparameters were set as follows: batch size to 30, learning rate to 0.01, the number of epochs to 80, an exponential learning rate scheduler with γ set to 0.999. Twenty training runs for each subject, were performed, in order to better evaluate the stability of the models training and the error trend along the epochs. The total number of parameters of the vEEGNet-ver3 is 4, 992 and the state dictionary (i.e., including all parameter weights) is 40kB-weight. The total number of parameters of the hvEEGNet model is 8,224, with 5, 456 of them to define the encoder, and the remaining 2, 768 for the decoder. Note that the higher number of parameters in the decoder is due to the sampling layers that operate on the three different latent spaces. The state dictionary of the parameter weights is about 56kB.

Finally, for the kNN algorithm for outliers detection (see Section 3.6), we employed the well-known *knee method* in the implementation given by the kneed python package (Satopaa et al., 2011) to find the *threshold distance* to actually mark some samples as outliers.

To foster an *open science* approach to scientific research, we made our code available on GitHub (at: https://github.com/jesus-333/Variational-Autoencoder-for-EEG-analysis).

### 3.8 Performance evaluation

In this work, we evaluated our models in a within-subject scenario (Zancanaro et al., 2021), only. Cross-subject evaluations, even though possible, are left for future developments as they deserve an entire new campaign of experiments and analyses.

The evaluation was carried on based on two different approaches: first, visual inspection of the reconstructed data in both the time and frequency domains (with the most convenient frequency range selected figure by figure); second, the quantification of the average reconstruction quality using the normalized DTW similarity score, as defined in Equation 5.

For visual inspection, we compared in a single plot the time domain representations of the original EEG segment and its corresponding reconstructed one. Also, we computed the Welch's spectrogram (Welch, 1967) (in the implementation provided by the Python Scipy package, available at: https://docs.scipy.org/doc/scipy/reference/generated/scipy.signal.welch.html) with the following parameters: Hann's window of 500 time points with 250 time points overlap between consecutive segments.

Then, to train and test our models (both vEEGNet-ver3 and hvEEGNet), we inherited the same split proposed by Blankertz et al. (2007): for every subject, 50% data were used for the training and the remaining 50% (i.e., a later experimental session) for the test. Furthermore, we applied cross-validation using 90% of the training data for the actual models' training and 10% for the validation. With the aim of investigating the training behavior of our models w.r.t. the particular input dataset, we repeated 20 training runs for each subject (i.e., each run started from a different random seed, thus ensuring a different training/validation split in the overall training set). This allowed us to provide a more robust evaluation of the training curve along the training epochs. We reported the models' performance in terms of descriptive statistics (mean and standard deviation across multiple training runs) of the reconstruction error along the training epochs (i.e., in other words, the training time). To note, in some rare cases where the loss function's gradient could not be minimized, we excluded those training runs from our final evaluation and training visualization.

Finally, the reconstruction ability of our models, after proper training (i.e., 80 epochs), was evaluated on the test set, too, by means of the same normalized DTW similarity score.

## 4 Results and discussion

### 4.1 Dataset

Dataset 2a of BCI Competition IV (Blankertz et al., 2007) was downloaded using the MOABB tool (Jayaram and Barachant, 2018) and it is composed by the 22-channel EEG recordings of nine subjects while they repeatedly performed four different motor imagery (MI) tasks: imagination of the movement of the right hand, left hand, feet or tongue. Each *repetition* consists of about 2 s fixation cross task, where a white cross appeared on a black screen and the subject needed to fix it and relax (as much as possible). Then, a 1.25 s cue allowed the subject to start imagining the required movement. The cue was displayed as an arrow pointing either left, right, up, or down, to indicate the corresponding task to perform, i.e., either left hand, right hand, tongue, or feet MI. MI was maintained until the fixation cross disappeared from the screen (for 3 s). A random inter-trial interval of a few seconds was applied (to avoid subjects habituation and expectation). Then, several repetitions of each type of MI were required to be performed. The order to repeat the different MI tasks was randomized to avoid habituation. The timeline of the experimental paradigm can be found in the original work by Blankertz et al. (2007).

A total number of 576 trials (or, repetitions) was collected from each individual subject. The EEG data were recorded with a sampling frequency of 250 Hz and the authors filtered the data with a 0.5 − 100*Hz* band-pass filter and a notch filter at 50 Hz (accordingly to the experimental records associated with the public dataset). We kept these settings as they were, to be in line with the literature (Lawhern et al., 2016) and to be consistent with our previous studies (Zancanaro et al., 2021, 2023a,b).

As explained in Section 3.8, we adopted the pre-defined 50/50 training/test split on the dataset and thus, for each subject, we obtained 260 EEG segments for the training set, 28 for the validation set, and 288 for the test set. To note, the dataset was perfectly balanced in terms of stratification of the different subjects in all splits.

**Figure 3:**
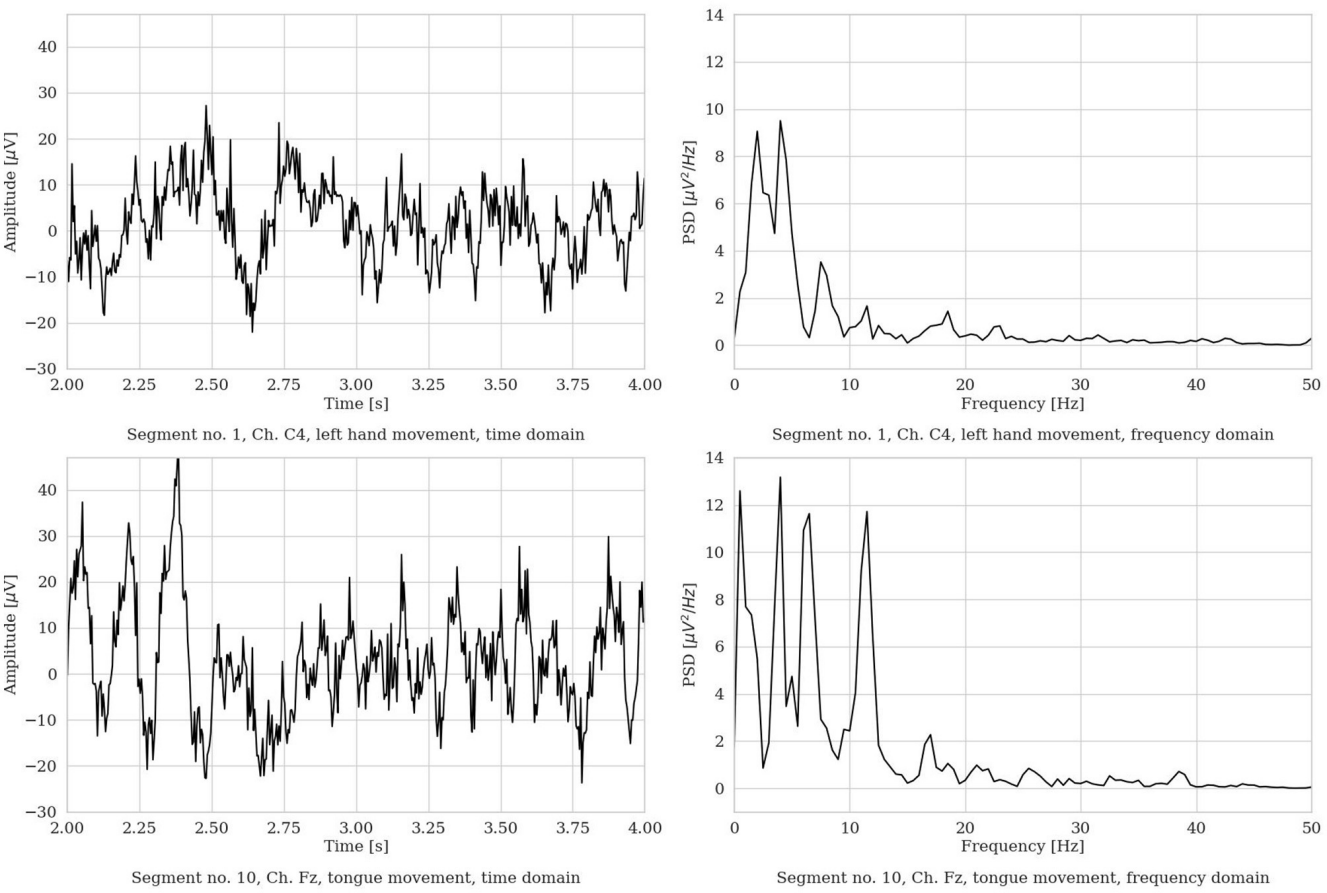
Two representative EEG segments from the Dataset 2a from S3 (time range limited from 2 to 4 s, frequency range limited to 50 Hz). **(Left)** Time domain representation. **(Right)** Frequency domain representation.

We performed segmentation and, for each MI repetition, we extracted a *4s* (22-channel) EEG *segment*. The piece of EEG was selected in the most *active* MI part of the repetition, i.e., from 2 to *6s*, in order to isolate the most apparent brain behavior related to the MI process. Figure 3 shows an example of two raw EEG signals, represented both in the time domain and in the frequency domain (with the frequency range limited to 50 Hz for visualization purposes). To note, to improve visualization in the time domain, the signals are shown in the limited time range from 2 to *4s*. However, in the frequency domain, the entire 4 s segment was used to compute the power spectrum (via Welch method, as described in Section 3.8). Then, a total of 1, 000 time points are included in each EEG segment.

### 4.2 Reconstruction performance

In this section we show the performance of vEEGNet-ver3 and hvEEGNet, and we discuss to what extent the new loss function (with the DTW contribution) and the hierarchical architecture influenced the reconstruction performance.

**Figure 4:**
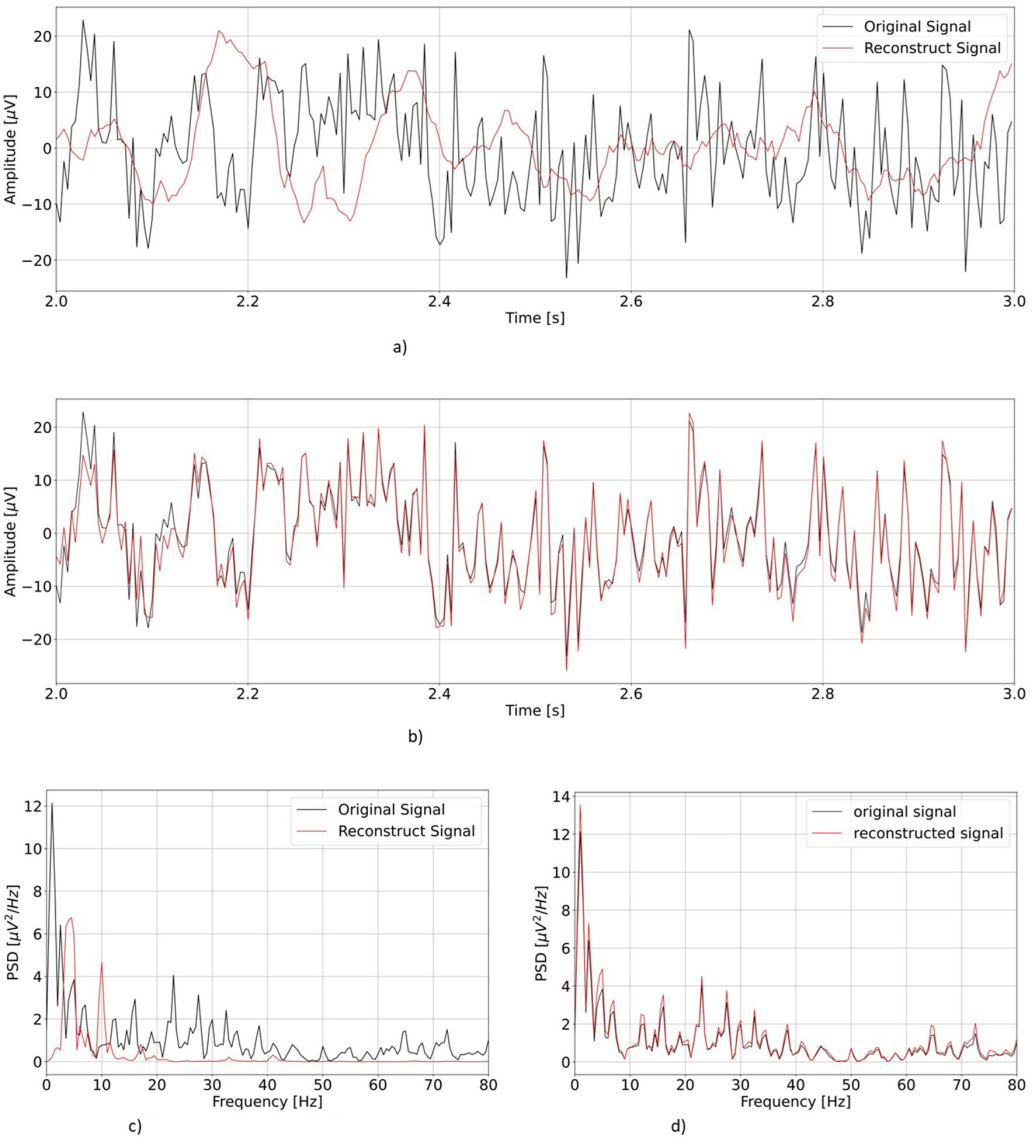
Comparison of the reconstruction performance in the time and frequency domain (in the test phase) in a representative subject (S3), task repetition [no.1, corresponding to **LH** MI], channel (C3). Time range was limited from 2 to 3 s, frequency range extended to 80 Hz. **(A)** Reconstruction of vEEGNet-ver3. **(B)** Reconstruction with hvEEGNet. **(C)** Frequency domain vEEGNet-ver3. **(D)** Frequency domain hvEEGNet.

First, we visually inspect the output from our two models. Figure 4 shows a representative example of one EEG segment as reconstructed by vEEGNet-ver3 and hvEEGNet, respectively, in both the time and the frequency domain (with the frequency range extended to 80 Hz for visualization purposes).

As it can be observed, hvEEGNet is much better in reconstructing the EEG segment, and this can be very clearly appreciated in both domains. However, it is worth mentioning that vEEGNet-ver3 brought a large improvement w.r.t. its previous versions [i.e., vEEGNet-1 (Zancanaro et al., 2023a) and vEEGNet-2 (Zancanaro et al., 2023b)] as well as to other recently proposed architectures in the literature (Bethge et al., 2022). In fact, from our previous work (Zancanaro et al., 2023a), we noticed that the model trained with a reconstruction error based on MSE was capable of reconstructing slow components, only, while now with vEEGNet-ver3 the reconstructed signal has a much broader spectrum, with higher frequency components. Therefore, we can conclude that our choice to train the models using a loss function where the reconstruction error is quantified by the DTW made a significant improvement. Nevertheless, we can also infer that the hierarchical architecture has a relevant influence in the ability of the model to reconstruct the signal with high-fidelity, as one might expect from the literature on VAEs as applied to reduce blurry effects in the reconstructed images (Vahdat and Kautz, 2021). To confirm these promising results, an extensive study on the generalization ability of hvEEGNet on other public datasets (e.g., Zhou et al., 2016) is ongoing, with preliminary encouraging outcomes (not reported for space constraints).

To more systematically compare the results from the two architectures, we filled Table 3 with all subject-wise performance of both models, after training (i.e., at the 80-th epoch) and in the test phase. Here, the mean values represent the average across channels and repetitions of the *normalized* DTW similarity score between every original EEG segment and its corresponding reconstructed one. Whereas, the standard deviation values were computed as the standard deviation of all mean values obtained by averaging across repetitions, only. The *grand*-average and the *grand*-standard deviation (i.e., the last two rows of the Table 3) are the mean and the standard deviation, respectively, taken across (the mean values of) all nine subjects. As we can observe, hvEEGNet largely outperforms vEEGNet-ver3 in all subjects, both during training and during test. It is worth noting that the data coming from two individuals, i.e., S2 and S5, resulted as particularly difficult to be reconstructed for both architectures. Later, we will deepen the investigation of these two cases providing a reasonable explanation for this problem.

**Table 3:** Average (± standard deviation) reconstruction error for vEEGNet-ver3 and hvEEGNet, as expressed in terms of *normalized* DTW similarity score.

	**vEEGNet-ver3**		**hvEEGNet**	
**Subject id**.	**Train**	**Test**	**Train**	**Test**
1	18.41 ± 6.26	22.99 ± 22.52	1.16 ± 0.36	2.3 ± 1.84
2	18.06 ± 8.88	128.05 ± 162.16	1.7 ± 0.81	60.81 ± 65.84
3	48.34 ± 14.19	41.35 ± 34.16	1.87 ± 0.62	4.96 ± 5.81
4	18.1 ± 21.66	18.01 ± 12.51	3.59 ± 7.44	1.51 ± 1.13
5	17.38 ± 15.16	49.09 ± 9.92	1.01 ± 0.45	15.67 ± 3.95
6	32.92 ± 13.28	29.01 ± 12.49	1.76 ± 0.72	1.87 ± 0.61
7	13.49 ± 3.67	12.37 ± 2.85	1.02 ± 0.28	0.9 ± 0.33
8	42.13 ± 21.19	48.5 ± 12.36	4.07 ± 1.61	5.46 ± 1.65
9	36.45 ± 21.33	33.87 ± 8.12	2.01 ± 1.23	1.91 ± 0.57
AVG	27.25	42.58	2.02	10.6
STD	13.96	30.79	1.5	9.08

Average and standard deviation were taken across repetitions.

**Figure 5:**
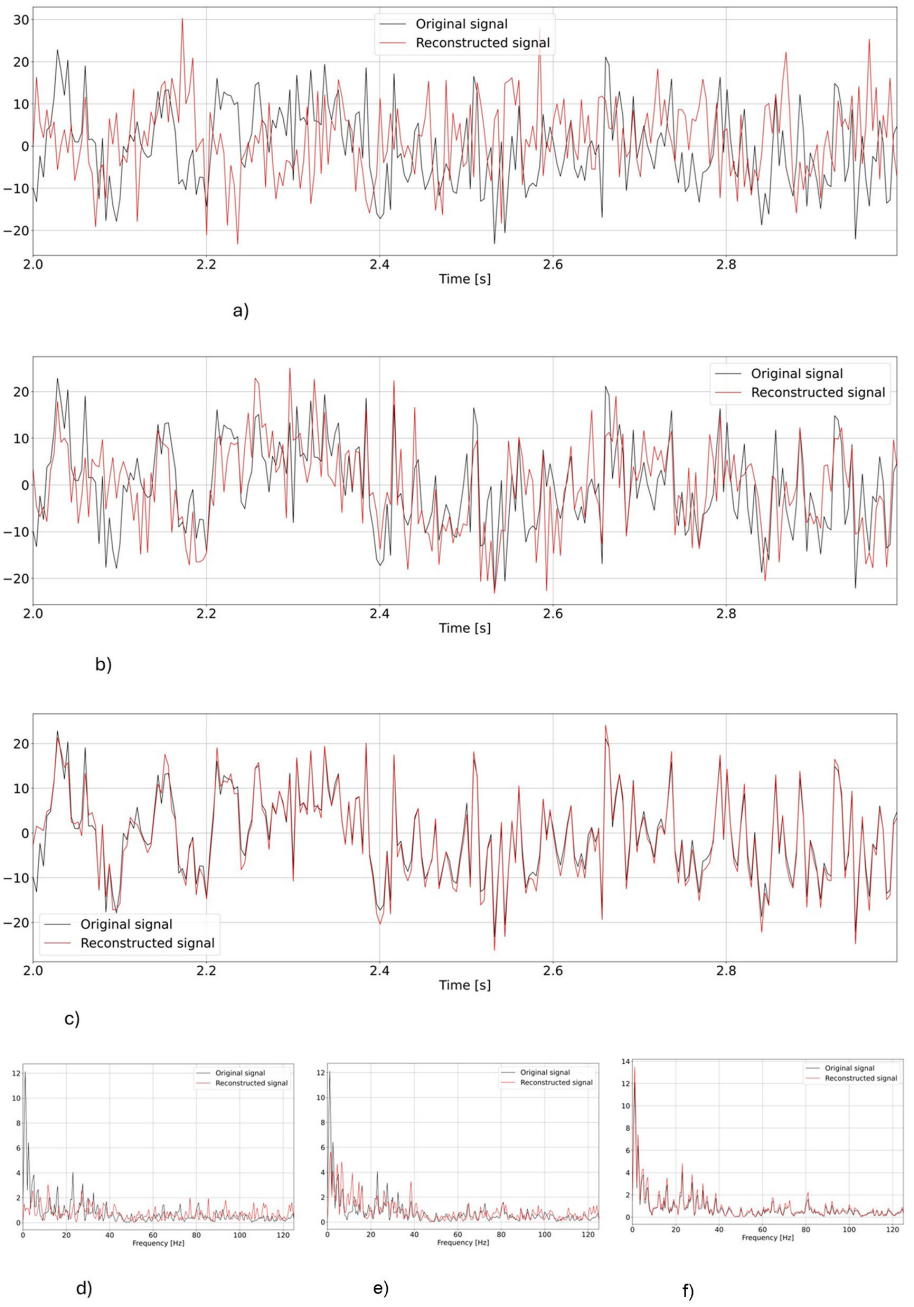
Reconstruction as obtained at different points of the hierarchy both in time and frequency domains for one representative subject (S3), task repetition (no.1, corresponding to left hand MI), and channel (C3), i.e., the same as in Figure 4. The first three rows represent time domain reconstructions, the last row reports power spectra in the three different points of the hierarchy. **(A)** Time domain reconstruction at the output of z1. **(B)** Time domain reconstruction at the output of z2 (including information from z1). **(C)** Time domain reconstruction at the output of z3 (including information from z2 and z1). **(D)** Output from z1. **(E)** Output from z2. **(F)** Output from z3.

Computer vision literature has already shown that the hierarchical architecture made the VAE models able to generate more detailed images, i.e., more effective in learning and generating high frequency components (Razavi et al., 2019; Prost et al., 2022). Here, the use of more than one latent space seemed to have similarly allowed hvEEGNet to better learn the underlying distribution of the data, and consequently greatly improved the reconstruction performance. This is also confirmed by Figure 5, where it is possible to see how the contributions of the three different latent spaces influenced the reconstruction performance of the model.

As it can be observed, the *deepest* latent space (*z*_1_) can quite follow the original signal, has a similar dynamics (check also the power spectrum in Figure 5), but suffers from some time shifts and amplitude mismatches. Still, this result is better than the vEEGNet-ver3 output, even though sampling from *z*_1_ in hvEEGNet could have similarities with sampling from *z*_0_ in vEEGNet-ver3 (e.g., much faster components can be recovered from *z*_1_, but not from *z*_0_). Then, sampling from more *superficial* (i.e., detailed) latent spaces produces an increasingly better reconstruction quality: when sampling from *z*_2_ (including the effect from the deepest latent space *z*_1_), amplitude mismatches are less frequent compared to the previous case, and the power spectrum is very similar to the original one. Finally, when sampling from *z*_3_, the reconstruction is almost perfect, with minimal amplitude incongruences and time shifts.

However, we found cases where hvEEGNet dramatically failed in reconstructing the original EEG data. Also, there were cases in which the same number of training epochs was not enough for the hvEEGNet model to reconstruct a particular subject. These two issues are discussed in the following, with additional investigations.

### 4.3 Training behavior vs. training set: investigations on hvEEGNet

**Figure 6:**
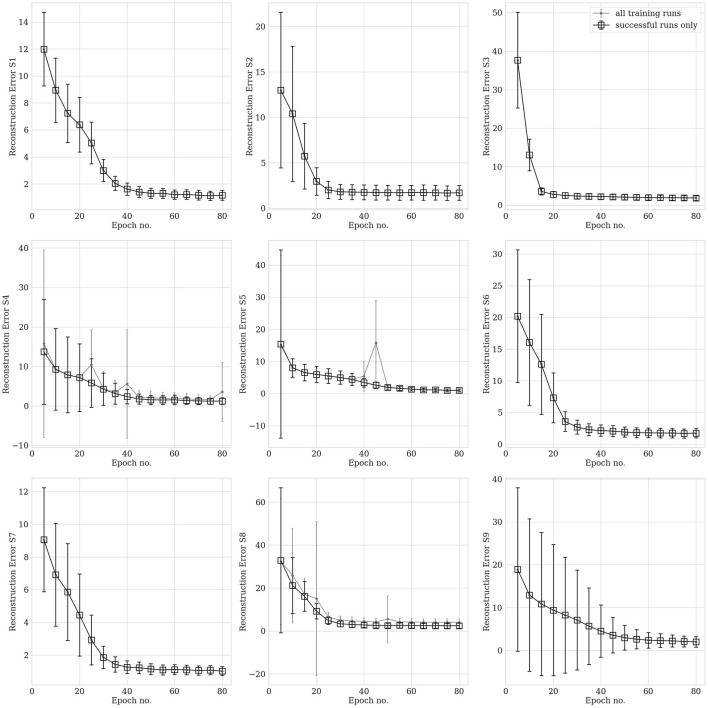
Reconstruction error (within-subject, across-EEG channels). Box markers show the average across multiple training runs, bars represent the standard deviation. Black is used to show results obtained with all training runs, while gray is when unsuccessful training runs (a few for three subjects out of nine, only) were removed from the analysis. Note that, for the sake of a better visualization, *y*-axes might have different ranges.

hvEEGNet should be trained until the DTW is small enough to guarantee optimal reconstruction. We performed several (about 20) training runs with 80 epochs each, to evaluate the statistical behavior of the model's training in different subjects. We also computed the average normalized DTW similarity score and its standard deviation across multiple runs and could show, for each subject, separately, the number of epochs at which that average is low enough and the standard deviation stabilizes, at the same time. Figure 6 displays the average (and standard deviation) DTW-based error for an increasing number of epochs for each subject during training. We observe that the DTW-based error clearly decreases as the number of epochs increases, as expected. Then, for all subjects, 80 epochs are enough to obtain high-fidelity reconstruction. However, we also clearly noted that the time (no. epochs) needed to reach that point highly varies from subject to subject. For example, S3 reaches an optimal model very rapidly, in about 15 epochs: we can see that the training of an hvEEGNet model starts with an average DTW error of 38 and a large standard deviation of 12, then it fastly decreases in its mean and variability, reaching a stable average of 5 and a very small standard deviation in 15 epochs. A completely different case is represented by S9: here, the average beginning error is smaller than the S3's one, but the standard deviation is much larger. Also, it takes much more - on average - to the model to adapt to this subject and reach a stable and optimal model (at about 60 epochs).

Therefore, we have just empirically proved that there is a relationship between the training time (i.e., the number of epochs needed to reach an optimal model) and the distribution of the input training set that cannot be overlooked (Gyori et al., 2022).

**Figure 7:**
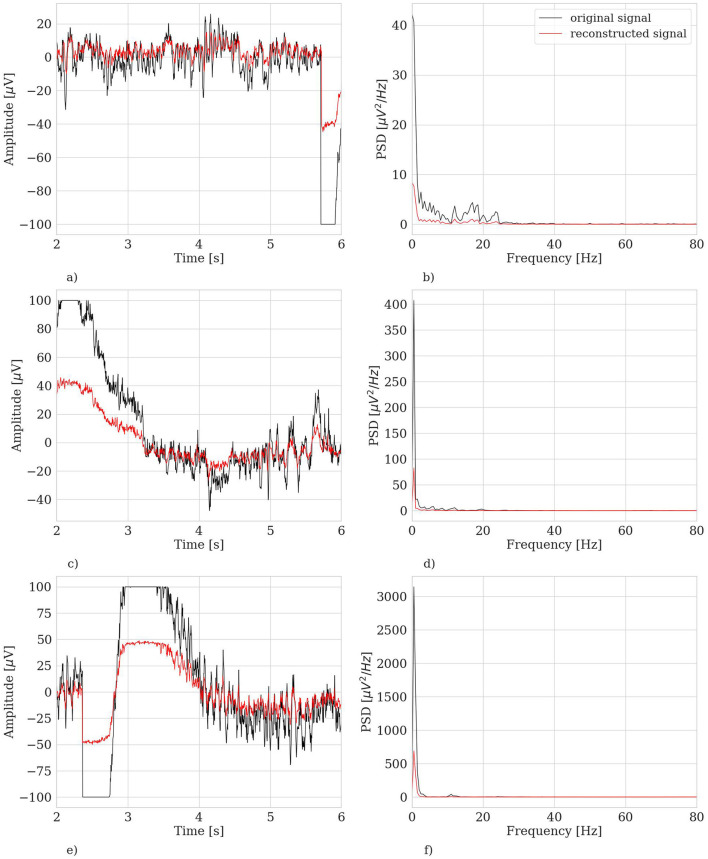
Example of saturated trials in the training set leading to very high DTW values. Left panels: time domain representation. Right panels: frequency domain representation. **(A)** S4, segment no. 180, ch. C3, time domain. **(B)** S4, segment no. 180, ch. C3, frequency domain. **(C)** S5, segment no. 221, ch. Cz, time domain. **(D)** S5, segment no. 221, ch. Cz, frequency domain. **(E)** S8, segment no. 82, ch. CP4, time domain. **(F)** S8, segment no. 82, ch. CP4, frequency domain.

Another relevant case to discuss is the dramatic fail of the hvEEGNet model in reconstructing some—rare—specific EEG segments. We found four anomalous training runs where the model failed, i.e., two for S4, one for S5, and another for S8. We further analyzed all segments in these three subjects and discovered that the model failure was due to problems of saturation that happened during the acquisition step of the EEG data (those segments had not been removed from the public available dataset). This, in turn, led the DTW score to assume extremely high values, i.e., the model to significantly fail the reconstruction. Figure 7 shows one example of EEG segment for each subject (S4, S5, and S8) where signal saturation was identified during the hvEEGNet model training. No matter where saturation occurs, i.e., soon or later in the segment, its effect on the model training is to dramatically increase the DTW-based error. These events, in turn, are responsible for that sudden increase of the standard deviation, as it can be noticed at epochs 25 and 40 for S4, at epoch 45 for S5, and at epoch 20 for S8. On the other hand, we also checked that the vast majority of the other S4, S5, and S8's segments led to DTW score values in a range similar to the other subjects. Thus, we decided to exclude those training runs where the hvEEGNet model suffered from the disruptive effect of acquisition saturation problems, namely unsuccessful training runs. For this reason, for S4, S5, and S8, Figure 6 shows the model training behavior along the epochs both with and without the unsuccessful training runs (gray and black line, respectively). Nonetheless, we cannot assess that saturation during acquisition is the only possible cause of training inaccuracy for the hvEEGNet model.

In fact, we can provide further insights that there is a correspondence between the training behavior and the quality of the input training set, thus highlighting the importance to preliminary evaluate the quality and the distribution of the input dataset. Besides, we also remind that the vast majority of the related work used this benchmark dataset as it is, as the input to a wide variety of DL models with no questions on the quality and distribution of the input data (Schirrmeister et al., 2017; Lawhern et al., 2016; Sakhavi et al., 2018; Li et al., 2019; Riyad et al., 2021; Zancanaro et al., 2021). All of them have shown a large variability in the classification results (i.e., classification of the different MI tasks), but there is no study—as far as the authors know—reporting a systematic investigation of the relationship between the models training and the characteristics of the input data.

In the following, we investigate the performance of the hvEEGNet model, when *sufficiently trained* (i.e., for a number of epochs that varies from subject to subject), and we explore its ability to identify anomalies as well as reconstructing clean EEG segments.

### 4.4 hvEEGNet as anomaly detector

Once the hvEEGNet model is properly trained, we can look into its ability to identify outliers. To be conservative, for all subjects, we searched for outliers in the test set with the hvEEGNet model trained for 80 epochs. As described in Section 3.6, we employed the kNN algorithm on the matrix $\bar{\text{E}}$ given by all values obtained by averaging the normalized DTW scores across the training runs for each pair task repetition-channel (subject-wise). Here, $\bar{\text{E}}$ is a 288 × 22 matrix. Then, we applied kNN over $\bar{\text{E}}$ to find out any possible outliers. We implemented the algorithm using the scikit-learn Python package (Pedregosa et al., 2011), with default settings and the number of nearest neighbors (*k*) equal to 15. We empirically found that 15 was a good trade-off between the stability of the results and the expected proximity among all samples in the dataset. Also, note that each sample of this matrix is characterized by 22 dimensions, and the kNN algorithm worked in such high-dimensional space to find proximity among points as well as outliers.

**Figure 8:**
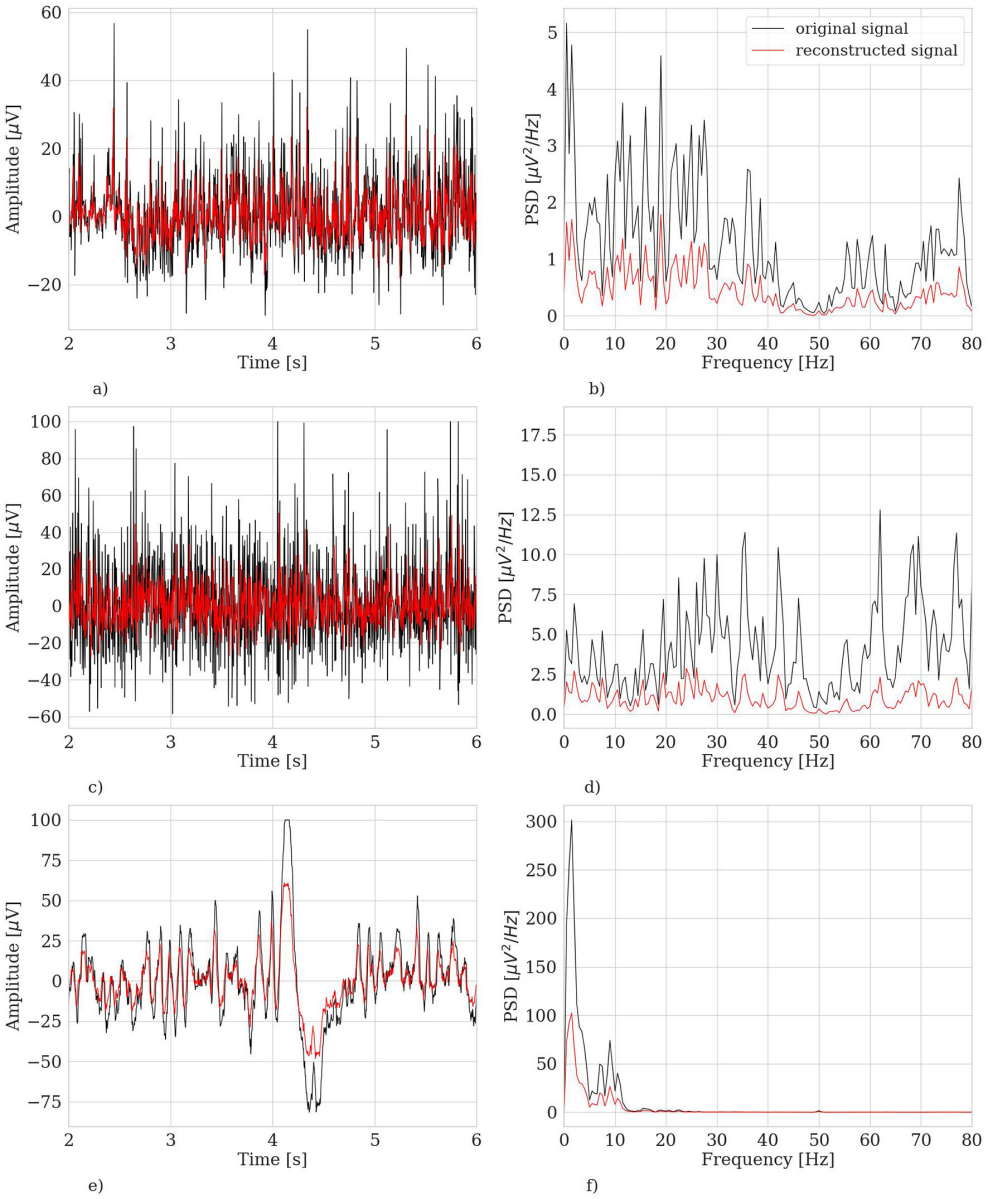
Three representative examples of EEG segments (belonging to the test set) marked as outlier by the hvEEGNet model extensively trained. Left panels: time domain representation. Right panels: frequency domain representation. **(A)** S2, segment no. 1, ch. FC3, time domain. **(B)** S2, segment no. 1, ch. FC3, frequency domain. **(C)** S4, segment no. 146, ch. C5, time domain. **(D)** S4, segment no. 146, ch. C5, frequency domain. **(E)** S9, segment no. 251, ch. C1, time domain. **(F)** S9, segment no. 251, ch. C1, frequency domain.

Figure 8 shows three representative examples of EEG segments that were marked as outliers by our extensively trained model. By visually inspecting them (in both time and frequency domain) and based on previous expertise (Cisotto et al., 2015) as well as well-established literature (Durka et al., 2003; Gao et al., 2010), we can easily confirm that those segments have a frequency characterization similar to a muscular artifact or eye blink activity.

**Figure 9:**
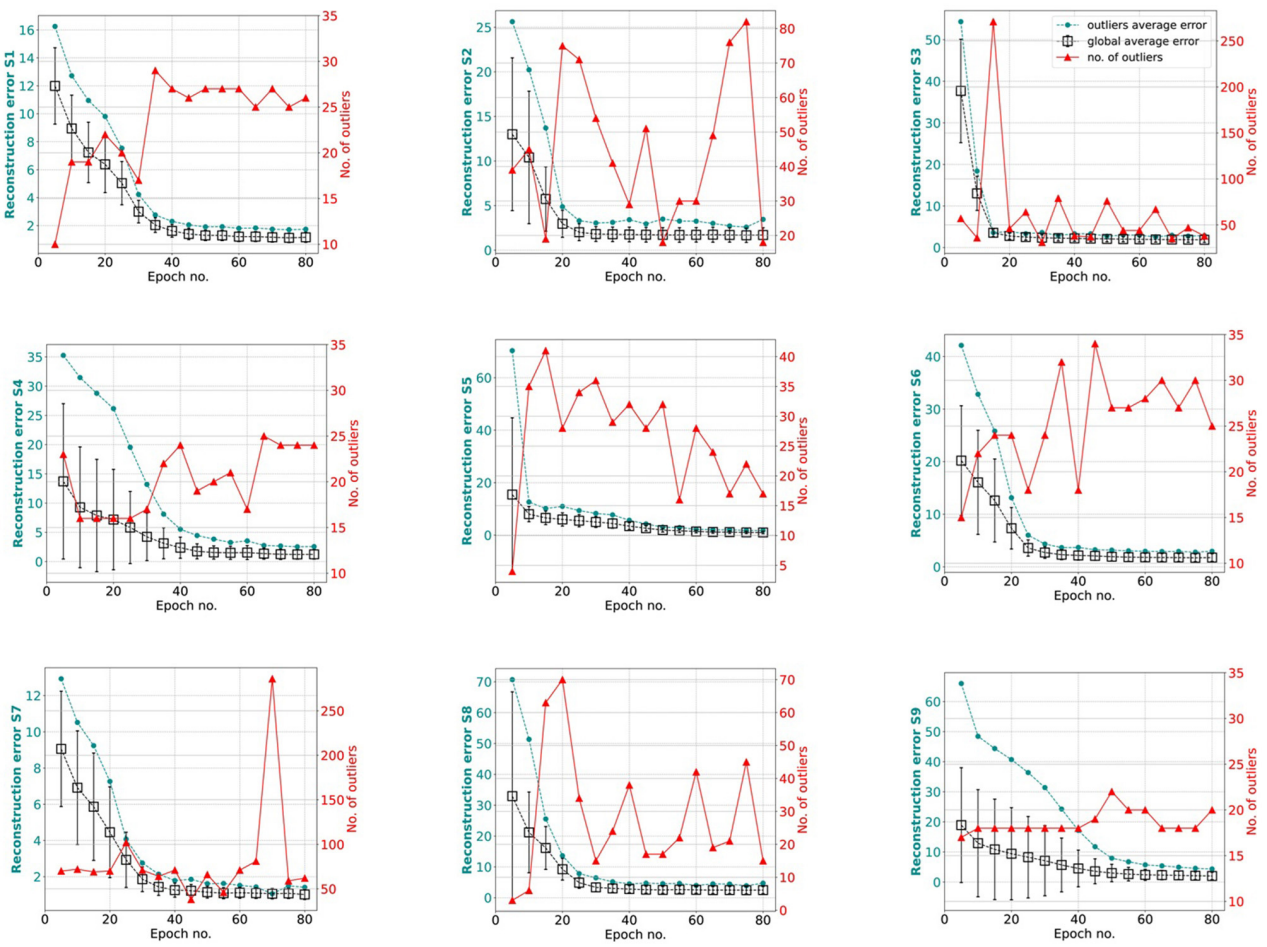
Relationship between training effectiveness and outliers identification ability w.r.t. individual subjects. Each panel reports the number of outliers (red line), the average error exclusively due to the outliers (dark green line), and the global average error of the whole training set (black line) for each subject (specified on the left *y*-axis), at each 5-epoch step during training.

However, Figure 6 has shown that the hvEEGNet model can reach very low average errors (with very small standard deviations) in a number of epochs typically lower than 80. Moreover, this time highly depends on the specific subject to analyze. To systematically investigate the relationship between the *training effectiveness* and the outliers identification ability w.r.t. individual subjects, we plotted Figure 9, where the global average error of the whole training set, the number of detected outliers, and the average error exclusively due to the outliers are reported for every subject, separately, at each 5-epoch step during training.

In all panels of Figure 9, i.e., for every subject, we can easily distinguish two phases in the training behavior: in the first part of the training, as expected, the average reconstruction error is progressively reduced (black line). This typically corresponds to a few outliers (red line) significantly contributing to the global average error (dark green line). In the second phase, i.e., after the model has reached a stable performance (in terms of average reconstruction error), the number of outliers starts to vary with the average global error and the outliers average error remaining quite small. This can be intuitively explained by the fact that the EEG segments are generally well-reconstructed and small variations on the error are enough to make the corresponding EEG segment to be considered an outlier by the kNN algorithm.

Therefore, we decided to deepen the investigation on the *transition point* to check if a subject-independent characterization of the training behavior can be obtained, and to verify the opportunity to stop the training at that point. First, we empirically defined the *transition point* as the number of epochs where the global average error showed the maximal slope (an elbow point), with low standard deviation, and the number of identified outliers was about to suddenly increase. For example, for S1 the transition point was identified at 30 epochs, while for S9 at 40 epochs. To note, the earliest transition point was found in S3 and S5 at 20 epochs, while the latest transition point was found in S4 at 45 epochs. Second, we re-evaluated the performance of our hvEEGNet model on the test set with the training stopped at the *transition point*. Table 4 reports the average (and standard deviation) reconstruction error at the subject-specific transition point. We can observe that the reconstruction performance are similar to the performance obtained for an extensively trained model (see Table 3 for the comparison). Thus, we can conclude that our hvEEGNet model could reach very high-fidelity reconstruction in a short time, lower than 30 minutes (approx. time needed to train the model for 50 epochs, as reported in Section 4.5).

**Table 4:** Average (± standard deviation) reconstruction error for hvEEGNet in the test set, with the model training stopped at the subject-dependent transition point.

		**Reconstruction error**	
**Subject id**.	**Transition point [epoch no.]**	**Train**	**Test**
1	30	3.0 ± 0.81	4.68 ± 3.55
2	45	1.73 ± 0.82	60.01 ± 65.89
3	20	2.79 ± 0.79	3.58 ± 1.53
4	45	2.18 ± 1.76	2.19 ± 1.71
5	20	5.97 ± 2.5	36.29 ± 6.44
6	25	2.01 ± 0.95	3.37 ± 1.02
7	30	1.86 ± 0.68	1.42 ± 0.43
8	30	5.06 ± 1.92	6.49 ± 1.82
9	40	4.51 ± 6.1	3.58 ± 0.8

**Figure 10:**
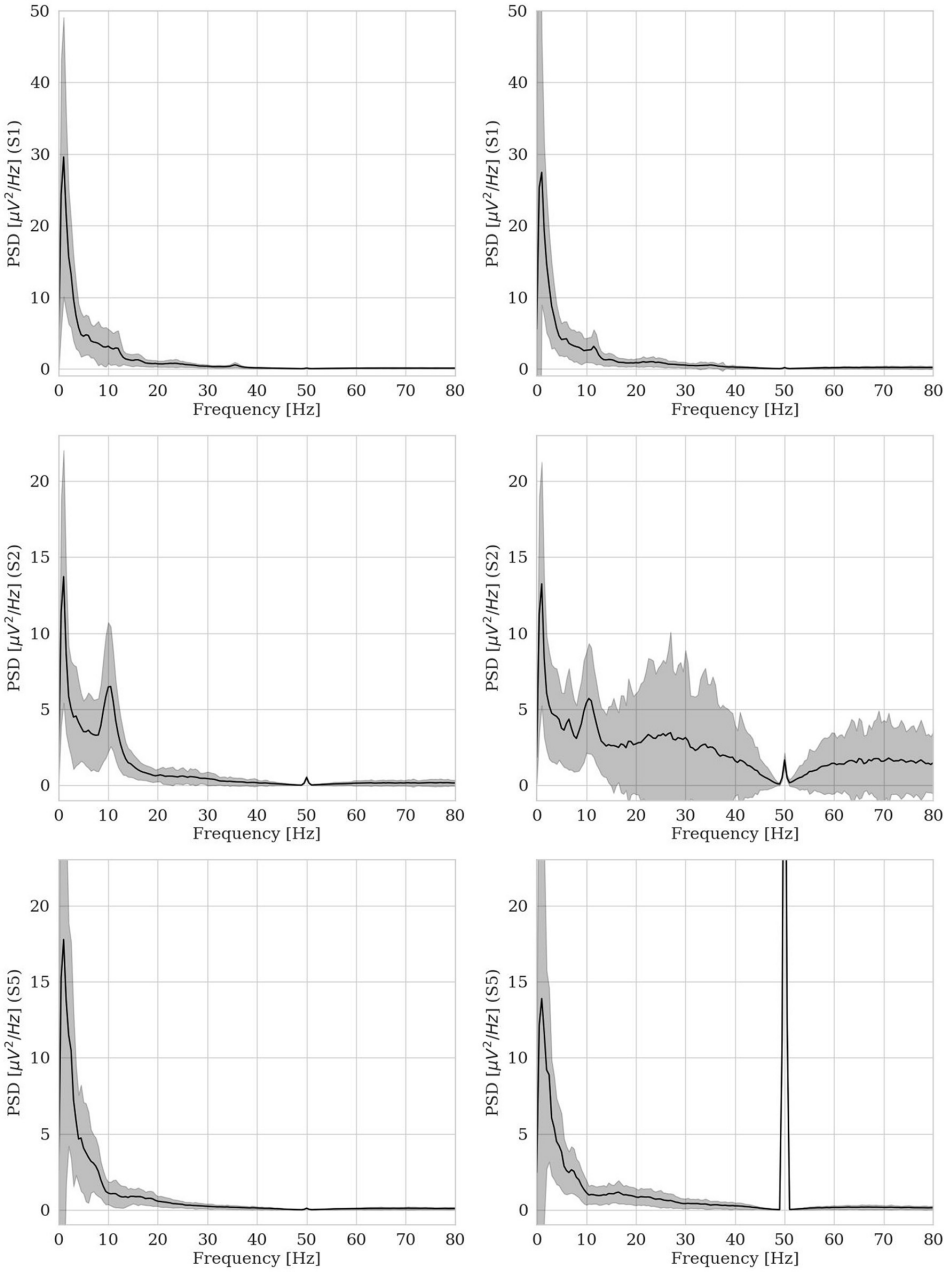
Average power spectra for training set (panels on the left side) and test set (panels on the right side) for three subjects (S1, S2, and S5) at channel Cz.

Usually, as already discussed in Section 2, when using autoencoder architectures to identify outliers, the model is trained on *normal* data (Ortiz et al., 2020) and anomalies result from the model's largest errors (Pang et al., 2021). Anyway, in more ecological acquisition scenarios (Muharemi et al., 2019) and, frequently, when the human is *in-the-loop* (Straetmans et al., 2022), anomaly detectors can be successfully trained on a mixture of clean and noisy data, too (Al-amri et al., 2021). Anyway, for EEG data, *normality* cannot be easily defined and it is quite challenging to ensure a dataset to be anomaly-free. For example, this public dataset was supposed to be fully *normal*, including a group of 9 healthy subjects, acquired via a research-grade EEG equipment, thus providing high data quality. Therefore, one might have expected to be able to build a robust anomaly detector based on this dataset. However, we showed that other kinds of *anomaly* are present and have been found by our hvEEGNet model: e.g., artifactual data, that affect the training set and the test set at different rates, thus making challenging the design of a traditional anomaly detector on these data. To support our claim and to deepen the investigation on those subjects having an out-of-normality distribution (i.e., S2 and S5, as already mentioned in Section 4.2), we provide Figure 10. It shows the average power spectra for the training set and the test set, separately, for three subjects at channel Cz. By inspecting this figure (and all other power spectra, not reported for space compactness), we realized how S2 and S5 are the only two individuals whose test sets were significantly different from all other data of this dataset. More specifically, we found out that the test set of S2 and of S5 (but not their training sets) are highly corrupted by noise and (muscular) artifacts. In fact, it is well-known (Buzsaki and Draguhn, 2004) that the typical power spectrum of a clean EEG acquired from a healthy subject follows a 1/*f* shape, with other relevant components (contributing as visible peaks) with center-of-band at about 10 Hz (the α band) and 20 Hz (the β band, generally less visible). In its upper panels, Figure 10 shows an example of clean dataset (from S1). Whereas, the lower panels report the power spectra of S2 and S5. It was decisive to visualize these spectra to realize that S2 has a *normal* (average) power spectrum in his/her training set, while a highly noisy power spectrum in his/her test set. Furthermore, it could be easily recognized that the large power contribution in other frequency ranges (e.g., higher than 50 Hz) is possibly due to muscular activity that was simultaneously recorded by the EEG electrodes during the test session (Chen et al., 2019). A similar situation was found for S5: again, all data coming from the test set were clearly corrupted by the 50 Hz power supply. We might only guess that, for some reason, the notch filter at 50 Hz (see Section 4.1) was not actually applied for this subject during the second recording session, i.e., the test session.

This finally explained why our hvEEGNet model dramatically failed at reconstructing S2 and S5 in their test sets, while keeping very satisfactory performance in the training phase. Furthermore, this might also motivate why the large majority of the related work classifying (i.e., with DL models, at least) this public dataset *always* found the worst results on S2 and S5 (Zancanaro et al., 2023a).

### 4.5 Computational complexity

Finally, we provide some reference measurements of the time spent in training and inference by our hvEEGNet model. We measured a training time, for each subject, of ~5 min for 10 epochs, on hardware freely available on Google Colab. This implies that a training run of 80 epochs for a single subject approximately takes 40 min. Note that the DTW is computationally heavier than MSE, thus increasing the training times. On the other hand, we proved that a DTW-based loss function leads to a significantly lower number of epochs needed for the training. Future improvements of our model might include the approximation of the DTW function itself with a neural network, as recently proposed by Lerogeron et al. (2023a,b).

Table 5 shows the inference time, i.e., the time the model used to encode and reconstruct 1, 10, 100, or 288 (all) EEG segments, respectively, using four different machines, namely CPU 1, GPU 1, CPU 2, and GPU 2. The details of the four machines are reported in the table's description. Results refer to the average (and standard deviation) time needed to perform 20 training runs.

**Table 5:** hvEEGNet inference time on four different machines.

**Batch size (no. of EEG segments)**	**CPU 1 (s)**	**GPU 1 (ms)**	**CPU 2 (s)**	**GPU 2 (ms)**
1	0.13 ± 0.05	8 ± 0.9	0.05 ± 0.003	3.24 ± 0.26
10	0.83 ± 0.13	23.3 ± 17.2	0.33 ± 0.03	10.45 ±1.58
100	5.91 ± 1.45	63.7 ± 10.3	5.02 ± 0.49	62.15 ± 4.95
288 (all)	10.69 ± 1.87	182.7 ± 30.1	14.73 ± 0.44	179.16 ± 18.22

CPU 1, Intel(R) Core(TM) i7-10750H CPU 2.60GHz (DELL G15 Laptop, 2020); GPU 1, NVIDIA GeForce RTX 2070 (DELL G15 Laptop, 2020); CPU 2, Intel(R) Xeon(R) CPU 2.20GHz (Google Colab); GPU 2, NVIDIA Tesla T4 (Google Colab). Note that CPUs times are expressed in seconds, while GPUs times in milliseconds.

The present work still suffers from a number of limitations, e.g., the investigation of the balance between the different components of the training loss and their impact of the training course and quality. Their investigation and solution have been left to further studies in favor of a few relevant take-home messages that can be robustly supported by the results available so far. As one of many possible future perspectives, hvEEGNet will be tested on different EEG datasets, including different types of anomalies, to prove its generalizability and the extent to which it can identify either artifactual or pathological EEG data.

## 5 Conclusions

Reconstructing a multi-channel EEG dataset with high-fidelity is a challenging task, given the complex dynamics of the EEG signals and the large inter-subject variability. Some fundamental challenges still need to be addressed, as our analysis of the SOTA revealed. Previous works have shown: (1) either high-fidelity reconstruction of *single-channel* EEG signals, or poor-quality reconstruction of *multi-channel* datasets, (2) a lack of focus on the reconstruction task (in favor of classification or anomaly detection tasks), even if the architectures employed could offer this possibility, and (3) no systematic investigation on the impact of the input data quality and distribution on the training of DL models (w.r.t. its effectiveness and the introduction of biases).

In this paper, we present a novel DL model, called *hvEEGNet*, designed as a hierarchical variational autoencoder with encoder/decoder modules inspired by the popular EEGNet architecture, and a new loss function (based on DTW) to effectively and fastly train the model. We tested *hvEEGNet* on the benchmark *Dataset 2a - BCI Competition IV*, where a 22-channel EEG data were collected from 9 subjects repeatedly performing motor imagery. We proved that the hierarchy is necessary to recover *multi-channel* EEG information (we compared *hvEEGnet* with *vEEGNet-ver3*), as well as our choice of DTW for the training was critical to significantly improve the reconstruction performance. This model is able to achieve high-fidelity reconstruction of multi-channel EEG signals in very short times (a few tens of epochs), compared to models at the SOTA and despite the small size of the dataset. Moreover, results are consistent across all subjects and repetitions. Then, we investigated the relationship between reconstruction fidelity and the training duration (in number of epochs) across different subjects, showing that the model can be trained even faster for some of them. Finally, using *hvEEGNet* as anomaly detector, we precisely spotted some corrupted data in the benchmark dataset (e.g., corruption by saturation during the acquisition phase) and never highlighted before. This, in turn, might explain that particular trend in the literature that always shows poor results, regardless the DL model employed, for those subjects in the dataset most impacted by the corruption. Therefore, *hvEEGNet* might find an important application to reduce the time spent by the domain experts to label clean/artifactual samples in large EEG datasets. This function of our model might also be useful in Internet-of-Medical-Things environments (Munari et al., 2023; Anders and Arnrich, 2022), where time-series data come from portable devices, typically more prone to artifacts and corruption.

This work opens new fundamental research questions about (1) the effectiveness and possible biases during training of DL models applied to EEG, and (2) the need for a systematic approach to evaluate the quality and the distribution of the input data to enable the effective training of such models. In the future, hvEEGNet and the proposed investigation methods could be applied to other EEG datasets [even including complex pathological conditions (Watorek et al., 2024)] and to other multi-channel time-series (e.g., ECG) to help in the modeling of complex dynamic systems, not limited to neuroscience. Our current approach as well as future perspectives intend to adhere to the best scientific methodological practices of new AI methods applied to the medical domain, in line with Cabitza and Campagner (2021).

## Data Availability

Publicly available datasets were analyzed in this study. This data can be found here: https://www.bbci.de/competition/iv/#dataset2a.
